# The tigecycline resistance mechanisms in Gram-negative bacilli

**DOI:** 10.3389/fcimb.2024.1471469

**Published:** 2024-11-20

**Authors:** Zhiren Wang, Henan Li

**Affiliations:** ^1^ Department of Clinical Laboratory, Peking University People’s Hospital, Beijing, China; ^2^ NHC Key Laboratory of Systems Biology of Pathogens, Institute of Pathogen Biology, Chinese Academy of Medical Sciences and Peking Union Medical College, Beijing, China

**Keywords:** Gram-negative bacilli, tigecycline, tigecycline resistance, resistance mechanism, resistance regulation

## Abstract

Tigecycline, hailed as a pivotal agent in combating multidrug-resistant bacterial infections, confronts obstacles posed by the emergence of resistance mechanisms in Gram-negative bacilli. This study explores the complex mechanisms of tigecycline resistance in Gram-negative bacilli, with a particular focus on the role of efflux pumps and drug modification in resistance. By summarizing these mechanisms, our objective is to provide a comprehensive understanding of tigecycline resistance in Gram-negative bacilli, thereby illuminating the evolving landscape of antimicrobial resistance. This review contributes to the elucidation of current existing tigecycline resistance mechanisms and provides insights into the development of effective strategies to manage the control of antimicrobial resistance in the clinical setting, as well as potential new targets for the treatment of tigecycline-resistant bacterial infections.

## 1 Introduction

Antimicrobial resistance is one of the most significant public health problems of our time. The Centers for Disease Control and Prevention (CDC) and the World Health Organization (WHO) have classified antibiotic-resistant pathogens as an imminent threat to human health (De Oliveira et al., 2020). The increased use of antimicrobial agents has led to an increase in the incidence of multidrug-resistant (MDR) or extensively drug-resistant (XDR) bacterial pathogens, which in turn has resulted in a rise in the number of prolonged hospitalizations, morbidity, and mortality cases. This has placed a significant economic burden on the healthcare system (Yaghoubi et al., 2022). Tigecycline, a tetracycline derivative, and colistin, a polymyxin, have been regarded as the “last line” of treatment for infections caused by MDR Gram-negative bacteria. However, the emergence of resistance has aroused widespread concern in recent years (Peng et al., 2022a).

Tetracyclines represent a class of compounds with a broad spectrum of antimicrobial activity, including Gram-positive and negative bacteria, spirochetes, intracellular bacteria, and parasites (Grossman, 2016). Tetracyclines encompass a diverse range of agents, from the naturally synthesized tetracyclines and chlortetracycline, which were introduced for medical use in the 1940s, to the second-generation semisynthetic derivatives doxycycline and minocycline, and the novel third-generation semisynthetic derivative tigecycline (Nguyen et al., 2014). In addition to the previously mentioned tetracyclines, new semisynthetic derivative omadacycline, and fully synthesized derivative eravacycline have been developed recently (Deolankar et al., 2022). These novel derivatives offer enhanced efficacy over first- and second-generation tetracyclines against challenging MDR Gram-negative and positive pathogens, including bacteria with tetracycline-specific resistance mechanisms (Grossman, 2016).

Tigecycline represents a novel semi-synthetic glycylcycline, a 9-t-butylglycinamido derivative of minocycline (Petersen et al., 1999). It can reversibly bind to 16S rRNA in the 30S subunit of the ribosome upon entry into the bacterial, thereby blocking tRNA access to the A-site and inhibiting the protein transcription-translation process. It was first introduced into clinical use in the United States in 2005 and subsequently entered the clinical setting in China in 2011. Due to the presence of a large substituent at position 9, it forms a large steric hindrance that can overcome the two major determinants of tetracycline resistance caused by ribosomal protection proteins Tet(M) and Tet(O) as well as the active efflux pumps Tet(A) and Tet(K) (Pankey, 2005; Pournaras et al., 2016). Tigecycline exhibits high *in vitro* activity against a wide range of Gram-positive and negative aerobic and anaerobic bacteria, including *Staphylococcus aureus*, *Haemophilus influenzae*, *Neisseria gonorrhoeae*, *Enterococci*, *Clostridium* spp., *Enterobacteriaceae*, *Bacillus* spp., and others, except *Pseudomonas aeruginosa*, *Proteus* spp., and *Morganella* spp. (Pankey, 2005). In addition to its approved use for complicated skin and skin structure infections, complicated abdominal infections, and community-acquired bacterial pneumonia in adults, recent reports have demonstrated the efficacy of tigecycline in the treatment of severe *Clostridium. difficile* infections (Kechagias et al., 2020). Due to its superior ability to inhibit the methicillin-resistant *S.aureus*, vancomycin-resistant *Enterococci*, and carbapenem-resistant *Enterobacteriaceae*, tigecycline remains a valuable therapeutic option for the treatment of severe infections caused by MDR pathogens. The tigecycline resistance mechanisms in Gram-negative bacilli have been extensively studied, revealing a wide distribution of resistance mechanisms across various species, along with some mechanisms that are specific to particular species.

## 2 Resistance mechanisms associated with efflux pumps and regulatory genes

Among the mechanisms of tigecycline resistance, the efflux pump, particularly the resistance-nodulation-cell division (RND) type efflux pumps, is essential. Inherent resistance in *P. aeruginosa*, *Proteus mirabilis*, and *Morganella morganii* has been reported to be mediated by two RND efflux pumps, MexXY-OprD and AcrAB (Pournaras et al., 2016). The tigecycline resistance-related efflux pumps and the regulatory genes have been documented in **Table 1**.

**Table 1:** Specific information on efflux pumps and its regulatory genes.

Family	Location	Genes and Gene clusters	Regulatory genes		Related species
RND	Chromosomal	*acrAB/EF*	*ramA*	+	*E.cloacae, E. coli, K. pneumoniae, S. typhimurium, S. enterica, E. aerogenes, E. hormaechei*
			*ramR*	–	
			*soxS*	+	
			*soxR*	–	
			*rarA*	+	
			*acrR*	–	
			*marA*	+	
			*marR*	–	
		*oqxAB*	*soxS*	+	*E. cloacae, K. pneumoniae, S. enterica*
			*rarA*	+	
			*ramA*	+	
			*marA*	+	
		*adeABC*	*adeRS*	+	*A. baumanii*, *A. nosocomialis*, *A. pittii*
			*baeRS*	+	
			*soxR*	–	
		*adeFGH*	*adeL*	–	
			*soxR*	–	
		*adeIJK*	*adeN*	–	
			*soxR*	–	
		*sdeXY*	NA	NA	*S. marcescens*
		*smeDEF*	NA	NA	*S. maltophilia*
		*axyEF*	NA	NA	*A. xylosoxidans*
	Plasmidic	*tMexCD1-toprJ1*	NA	NA	*K. pneumoniae*, *Klebsiella spp.*
	Plasmidic, chromosomal	*tmexCD2-toprJ2 tmexCD3-toprJ3*	NA	NA	*R. ornithinolytica*, *Klebsiella spp.*
	Plasmidic	*tmexCD3-toprJ3*	NA	NA	*K. aerogenes*
	Plasmidic	*tmexCD4-toprJ4*	NA	NA	*K. quasipneumoniae, E. roggenkampii*
MFS	Plasmidic	*tet*(A)	NA	NA	*K. pneumoniae, A. baumanii, S. enterica*
	Plasmidic	*tet*(Y)	NA	NA	*A. baumanii*
	Chromosomal	*tet*(L)	NA	NA	*Campylobacter* spp.
Novel	Chromosomal	*kpgABC*	NA	NA	*K. pneumoniae*

+, positive regulation; -, negative regulation; NA, not applicable.

### 2.1 RND efflux pump AcrAB/EF

The RND efflux pump AcrAB has been documented to be associated with tigecycline resistance in different strains. One study conducted transposon-mutagenesis of tigecycline resistant clinical *Enterobacter cloacae* isolates to obtain susceptible mutants (Keeney et al., 2007). The results indicated that mutants had transposon insertions in the *acrA* or *acrB* genes, whereas the complementation of the *acrAB* cloning plasmid restored their resistance phenotypes. Further RNA blotting demonstrated that the *acrAB* transcription was elevated in all strains exhibiting reduced tigecycline susceptibility. The overexpression of *acrAB* was observed to be associated with an increase in the expression of the transcriptional regulatory gene *ramA*, suggesting that tigecycline resistance in *E. cloacae* is a consequence of RamA-mediated overexpression of the AcrAB efflux pump. Liu et al. also demonstrated that the overexpression of regulatory genes *ramA* and *soxS* was associated with heterogeneous resistance to tigecycline (Liu et al., 2019). It was observed that the AcrAB and OqxAB efflux pumps were notably overexpressed in carbapenem-resistant tigecycline heterogeneous resistant *E. cloacae*, in which the increased expression of their regulatory genes *ramA* and/or *soxS* was presumed to be a key factor in the heterogeneous resistance of tigecycline.

In *Escherichia coli*, it has been reported that the transcriptional activator MarA is associated with the overexpression of the AcrAB efflux pump (Keeney et al., 2008). The transcriptional analysis of homozygous clinical isolates isolated from the same patient revealed that the expressions of *marA*, *acrA*, *acrB*, and *tolC* were significantly increased in strains with higher tigecycline minimum inhibitory concentrations (MICs). Transposon mutagenesis was also employed to generate tigecycline susceptible mutants, with the analysis revealing that the majority of which were *marA* or *AcrB* inactivated. Further sequence analysis showed a single nucleotide insertion (354_355insC) in the open reading frame of the *marR* gene in *E. coli* with higher tigecycline MICs, suggesting that the overexpression of MarA and AcrAB caused by the loss of MarR function due to the frame-shift mutation might, in turn, reduce the susceptibility to tigecycline. Furthermore, the AcrEF efflux pump in *E. coli* has been documented to be associated with decreased tigecycline sensitivity (Hirata et al., 2004). A series of research has identified that the loss-of-function mutation in the protease Lon in *Klebsiella pneumoniae*, *E. coli*, and *Salmonella typhimurium* can also cause tigecycline resistance (Nicoloff and Andersson, 2013; Fang et al., 2016; Linkevicius et al., 2016). Lon is involved in the degradation of MarA, and its inactivation leads to the overexpression of MarA and increases the expression of the AcrAB efflux pump, which in turn may result in resistance.

The overexpression of AcrAB and AcrEF in *Salmonella enterica* also results in tigecycline resistance (Horiyama et al., 2011). Horiyama et al. investigated the tigecycline MICs of *S. enterica* strains that overexpress or delete efflux pump and regulatory genes. The deletion of *acrAB* was found to increase the susceptibility to tigecycline, while the complementation of both *acrAB* and *acrEF* would restore the MIC in the deletion strains. Both the overexpression of *ramA* and the deletion of the negative regulatory gene *ramR* decreased the susceptibility but remained unchanged in the *acrAB*-deletion strains, suggesting that AcrAB and AcrEF confer resistance to tigecycline in *S. enterica* with the regulation of RamA and RamR. Moreover, the overexpression of the AcrAB efflux pump, which is the result of frame-shift and deletion mutations in *ramR* and amino acid substitution mutations in *ramA*, is the primary cause of tigecycline resistance in *E. aerogenes* (Veleba et al., 2013). Decreased tigecycline susceptibility caused by *ramR* mutations has also been observed in *E. hormaechei* (Gravey et al., 2020). Gravey et al. found an *E. hormaechei* isolate developed tigecycline resistance in a hospitalized patient after treatment without any relevant resistance genes acquisition except for a 16 bp deletion in *ramR*, which resulted in the overexpression of RamA, AcrAB, and TolC, as well as the downregulated expression of pore protein OmpF. This indicated that partial deletion of the *ramR* can lead to the overexpression of RamA, which in turn causes the increased efflux of AcrAB-TolC and decreased antibiotic permeability through OmpF, collectively contributing to tigecycline resistance.

AcrAB overexpression associated with tigecycline resistance in *K. pneumoniae* is frequently accompanied by the increased transcription of *ramA* and the inactivation mutations of *ramR*, and overexpression of the global regulators *rarA* and *marA* (Bratu et al., 2009; Hentschke et al., 2010b; Veleba et al., 2012a; Roy et al., 2013; Sheng et al., 2014; Villa et al., 2014; Zhong et al., 2014; Wang et al., 2015; Fang et al., 2016). Sequence analysis revealed that approximately 83% of tigecycline non-susceptible *K. pneumoniae* carried mutations in *ramR* and/or local repressor *acrR*, including missense or nonsense mutations, insertions, and deletions in *ramR*, as well as amino acid substitutions and frame-shift mutations in *acrR*. The results of real-time quantitative PCR (RT-qPCR) demonstrated increased expression of the *acrB* in all resistant strains, indicating that the combination of RamR and AcrR mutations might be involved in the reduced susceptibility to tigecycline (Moghimi et al., 2021). Mutations in the *acrR* gene may also be related to tigecycline non-susceptibility. Through the antimicrobial susceptibility tests in the presence of the efflux pump inhibitor, Zhang et al. isolated an efflux pump-related tigecycline non-susceptible *K. pneumoniae* strain only with a frame-shift mutation in *acrR* caused by a 2 bp deletion, suggesting that the *acrR* gene mutation might be associated with reduced tigecycline susceptibility (Zhang et al., 2021c). Furthermore, mutations in the negative regulatory genes *ramR* and *soxR* were also found to be relevant with tigecycline heterogeneous resistance in *K. pneumoniae* (Zhang et al., 2021a). A heterogeneous resistance subpopulation was identified in tigecycline-sensitive *K. pneumoniae* by the disk diffusion method, in which mutations in *ramR* and *soxR* were found. The mutations would induce the expression of *ramA* and *soxS*, which in turn would cause the overexpression of AcrAB-TolC, thereby resulting in tigecycline heterogeneous resistance and resistance.

### 2.2 RND efflux pump OqxAB

OqxAB is also a class of RND efflux pumps that have been involved with tigecycline resistance. Previous study has investigated the expression of efflux pumps and regulatory genes in tigecycline-resistant *K. pneumoniae* by RT-qPCR (Zhong et al., 2014). The results presented that the susceptible strains exhibited higher expression levels for both *oqxB* and the regulatory gene *rarA* in OqxAB. For isolates with MICs up to 8 mg/L, the AcrAB-TolC efflux pump plays the most important role in tigecycline resistance, in contrast, both the AcrAB-TolC and OqxAB efflux pumps are required for isolates with MICs ≥ 16 mg/L. The overexpression of *rarA* in *K. pneumoniae* has been reported to upregulate the neighboring OqxAB efflux pump, resulting in tigecycline resistance (Veleba and Schneiders, 2012b). The knockout of another regulator *ramA* showed a slight increase in tigecycline susceptibility, further resistance screening revealed that the transcription of *oqxAB*, *acrAB*, *rarA*, and *marA* were significantly elevated in the resistant strains (Veleba and Schneiders, 2012b). This indicated that regulators RarA and MarA provide an alternative pathway for tigecycline resistance in *K. pneumoniae*. Chen et al. also found OqxAB efflux pump was associated with tigecycline heterogeneous resistance in *S. enterica* (Chen et al., 2017). The addition of the efflux pump inhibitor restored the susceptibility of tigecycline and reduced its accumulation in the cells, suggesting that the heterogeneous resistance was due to the overexpression of the AcrAB-TolC and OqxAB efflux pumps (Chen et al., 2017).

### 2.3 RND efflux pump Ade family

It has been reported that the overexpression of three RND efflux pumps, AdeABC, AdeFGH, and AdeIJK, is associated with tigecycline resistance in *Acinetobacter baumannii* (Coyne et al., 2011). AdeABC plays a significant role in the efflux of tigecycline, while the two-component system AdeSR, which contains a sensor kinase protein AdeS and a regulatory protein AdeR, regulates the transcription of the efflux pump. In contrast, AdeFGH is regulated by the LysR-type transcriptional regulator AdeL, and AdeIJK is regulated by the TetR transcriptional regulator AdeN. Many previous studies have found that the tigecycline resistance caused by elevated levels of efflux pump transcription may be related to amino acid substitutions or insertions of insertion sequences (ISs) in regulatory genes (mutation sites are shown in **Table 2**) (Coyne et al., 2010; Rumbo et al., 2013; Yoon et al., 2013; Yoon et al., 2015; Sun et al., 2016; Hua et al., 2021; Lucaßen et al., 2021a; Lucaßen et al., 2021b; Salehi et al., 2021). The most common insertion sites for IS*Aba1* are positions 371, 379, 422, and 430 of *adeS* and positions 52, 200, and 402 of *adeR*. Nevertheless, the tigecycline resistance resulting from the IS*Aba1* insertion at *adeS* is prone to instability during successive passages in the absence of tigecycline, which belongs to heterogeneous resistance, and can be reversed to a susceptible strain by additional insertion of IS*Aba1* into *adeR* (Jo and Ko, 2021).

**Table 2:** Common amino acid mutation sites in Ade efflux pumps and regulatory genes.

Protein	Amino acid mutation sites	Structural location	PMID
AdeS	G30D	periplasmic input domain of the sensor	19884373
	E51K	sensor domain	34170209
	A94V/G103D/N125K	HAMP linker domain	23587960/20554571/23939894
	R152K/T153M/T153A/T156M/D167N/D167A	near the DHp domain putative autophosphorylation site His-149	25805730/23587960/15328088/34817237/26488727/33760099
	the C487T nucleotide change led to a stop codon (Q163stop)	near the DHp domain putative autophosphorylation site His-149	23587960
	G186V	α helix of DHp domain	24939621/33691788/26850720/23939894
	H189Y	C-terminal of DHp domain	23587960/33691788
	I252S	near the N box in the catalytic domain	23587960
	I100N/E121K/L172P/F214L/N268H/S280A/Q281D/Y303F/G336S/Q339K	NA	23587960/33760099/33691788/23939894
	premature stop codon	NA	33760099
AdeR	E19D/D20N	phosphorylation site	20921306/33760099
	D26N	α1 helix	33760099/34817237
	P56S	phosphorylation site	23587960
	L192R	effector domain	23587960
	E219A	DNA binding domain	23587960
	A91V/A136V	signal receiver domain	20554571/24939621/25805730/33691788/23939894
	P116L	α5 helix	15328088
	D21V/A101T/I20V/L142I/E204K	NA	33760099/33691788
	nucleotide substitution in AdeR binding site	NA	33760099
AdeL	T319K/N334H	C-terminal region	20696879/25805730
	a thymidine insertion at position 981 led to a premature stop codon	C-terminal region	20696879
	V139G	putative signal recognition domain	20696879/23587960
	premature stop codon (Q326/Q332stop)	NA	25805730/23587960
	P125L/Q262R/C292G	NA	33760099/33691788
AdeN	premature stop codon at position 211	α9 helix of putative dimerization domain	22371895
	C584 deletion	NA	25805730
	P16T/L35R/A43P/G54S/G65D/N66Y/H111P/I112F/K141N/L173F	NA	33760099/23939894
	premature stop codon	NA	33760099

NA, not applicable.

The most prevalent and widely distributed mechanism identified is the inactivation of the AdeIJK repressor gene *adeN*, which may have been caused by premature stop codon resulting from ISs insertion or nucleotide deletion. In contrast, mutation or inactivation of the AdeABC regulatory system *adeRS* occurs less frequently but is often associated with higher tigecycline MICs (Lucaßen et al., 2021a). Lucaßen et al. have evaluated the effect of frequently reported amino acid substitutions on *adeB* expression, efflux activity, and tigecycline susceptibility through the construction of related knockout and complementary strains (Lucaßen et al., 2021b). The complementation of D26N mutant AdeR and T156M mutant AdeS into the *adeRS* knockout strain led to higher tigecycline MICs as well as a significant increase in *adeB* expression and antibiotic efflux. Conversely, the complementation with D21V mutant AdeR did not affect the susceptibility or efflux pump expression, indicating that amino acid substitutions D26N (AdeR) and T156M (AdeS) disrupted their regulatory functions and impacted the efflux. Another two-component regulatory system *baeRS* has also been shown to affect the tigecycline susceptibility in *A. baumannii* through the upregulation of *adeAB* (Lin et al., 2014). In addition to the aforementioned transcriptional regulators, the expression of *adeABC* can be overexpressed under low iron environments, indicating the potential for additional regulation of the efflux pump by iron (Modarresi et al., 2015).

The global regulator SoxR has been reported to increase tigecycline susceptibility by affecting the expression of efflux pumps (Li et al., 2017). Li et al. compared the molecular characterization between MDR and tigecycline susceptible *A. baumannii* strains and found that the frequency of the G39S mutation in SoxR was higher in MDR strains. Further overexpression of *soxR* revealed a minor decrease in tigecycline MIC and a reduction in the expression of the efflux pump genes *adeJ* and *adeG*, the small multidrug resistance family gene *abeS*, and the multidrug and toxic compound extrusion gene *abeM*, suggesting that SoxR might act as a negative regulator of efflux pump and contribute to tigecycline resistance in *A. baumannii*.

Moreover, the overexpression of the Ade efflux pump has been documented in *A. nosocomialis* and *A. pittii*. One study investigated the contribution of the RND efflux pump to tigecycline resistance in *A. nosocomialis*, focusing on the expression of the RND efflux pump and the *adeR* mutation (Yang et al., 2019). The RT-qPCR results revealed that tigecycline non-susceptible isolates exhibited a significantly increase transcription of *adeB* compared to susceptible ones, indicating that AdeABC was the major efflux pump related with tigecycline non-susceptible in *A. nosocomialis*. Further comparative sequence analysis identified five amino acid substitutions in AdeR and AdeS, including S16N and H56L in AdeS, D299N, T137N, and A220E in AdeR. The significant increases in the transcription of *adeB* and *adeJ* correlated with tigecycline resistance were also observed in tigecycline resistant *A. pittii* (Ding et al., 2022).

### 2.4 Other chromosome-localized RND efflux pumps

There are three intrinsic RND efflux pumps, namely SdeAB, SdeCDE, and SdeXY, in *Serratia marcescens*, whose efflux has been confirmed to be an important mechanism for tigecycline resistance (Hornsey et al., 2010). Hornsey et al. conducted the mutation screening by successive passaging under tigecycline pressure, resulting in the isolation of a mutant with a 256-fold increase in tigecycline MIC. The RT-qPCR analysis revealed that all three RND efflux pumps were overexpressed in the mutant, while the transcription of *sdeXY* was significantly higher than others. Further investigation of the constructed mutant indicated that the tigecycline MIC was significantly decreased when *sdeY* and the putative outer membrane component gene *hasF* were inactivated, suggesting that the overexpressed SdeXY-HasF pump was the possible reason for the tigecycline resistance in *S. marcescens*.

The overexpression of RND efflux pump SmeDEF is a predominant contributing factor for tigecycline resistance in *Stenotrophomonas maltophilia* (Blanco et al., 2019). During the experimental evolution, the tigecycline-evolved populations attained resistance through different mutational trajectories, with all initial mutations occurring in the SmeDEF repressor *smeT*, which might result in the overexpression of the efflux pump. An RND efflux pump, AxyEF-OprN, which mediates tigecycline efflux has also been identified in *Achromobacter xylosoxidan*s (Nielsen et al., 2019). Following targeted AxyEF by transcriptome analysis, the study knocked down *axyE* and found a decrease in tigecycline MIC, demonstrating that AxyEF-OprN might efflux tigecycline and interfere with tigecycline resistance.

### 2.5 Plasmid-mediated RND efflux pumps

Previous studies have indicated that RND efflux pump gene clusters on plasmids may contribute to tigecycline resistance. Lv et al. identified a novel RND efflux pump gene cluster, *tnfxB1-tMexCD1-toprJ1*, on plasmids from pan-resistant *K. pneumoniae* (Lv et al., 2020b). The overexpression of the cluster resulted in the elimination of the *in vitro* and *in vivo* accumulation of tigecycline in bacteria, suggesting that TMexCD1-TOprJ1 could mediate tigecycline efflux and generate resistance that might lead to tigecycline treatment failure. Plasmids with TMexCD1-TOprJ1 have been discovered in Asia, Europe, and North America, indicating a risk of global spread of tigecycline resistance. Furthermore, a plasmid co-localizing *tmexCD1-toprJ1* and the colistin resistance gene *mcr* has been reported in *K. pneumoniae*, which also carried an IncX3 plasmid with *bla*_NDM_ (Sun et al., 2020). The emergence of the mobile tigecycline and colistin resistance poses a substantial threat to public health, underscoring the urgent need for further global surveillance.

A novel plasmid-mediated RND efflux pump gene cluster, *tnfxB2-tmexCD2-toprJ2*, which exhibits high similarity to *tnfxB1-tMexCD1-toprJ1*, has been identified on the chromosome and plasmid of *Raoultella ornithinolytica* (Wang et al., 2021b). The overexpression of *tmexCD2-toprJ2* demonstrated an 8-fold increase in tigecycline MIC, while the genetic environment analysis revealed its translocated potential between the plasmids and chromosomes. This cluster has been reported in various *Klebsiella* spp., and novel related isoforms *tmexC1D1.2-toprJ1* and *tmexC2D2.2-toprJ2* have also been identified in *P. aeruginosa* and *Klebsiella spp*, indicating that this plasmid-mediated tigecycline resistance mechanism has already spread among isolates and improved detection is necessary to prevent the resistance crisis (Wang et al., 2021d; Sun et al., 2022).

In addition to *tmexCD1/2-toprJ1/2* and *tmexCD3-toprJ3*, which has been found in *Proteus* spp. and *P. aeruginosa* (Wang et al., 2021a), a fourth *tmexCD-toprJ*-like gene cluster, *tmexCD4-toprJ4*, has been identified in plasmids from *K. quasipneumoniae* and *Enterobacter roggenkampii* that mediates tigecycline resistance (Gao et al., 2022). The overexpression of *tmexCD4-toprJ4* resulted in increased tigecycline efflux and MICs. Gao et al. further found that *tmexCD4-toprJ4* could act synergistically with its upstream *tet*(A) to reduce the susceptibility. A recent epidemiological study of the clinical prevalence, genomic, and phenotypic characterization of *tmexCD-toprJ* has shown that among 7,517 clinical isolates collected in China, 48 isolates carried *tmexCD-toprJ* (0.64%), all of which were MDR and possessed other resistance genes simultaneously (Dong et al., 2022b). The presence of mobile elements in the genetic environment suggests that it may be capable of propagating among different species, prompting the necessity for monitoring and control of the further spread.

### 2.6 MFS efflux pumps

The major facilitator superfamily (MFS) efflux pumps represent another class of efflux pumps involved in tigecycline resistance. It has been demonstrated that the RND-type efflux pumps AdeABC and AdeIJK can synergize with the MFS efflux pump TetA to induce tigecycline resistance in *A. baumannii* (Foong et al., 2020). Foong et al. overexpressed *tet*(A) in the RND efflux pump knockout strains and detected decreased tigecycline MICs in tigecycline susceptible *adeAB* and *adeIJ* knockout strains, suggesting that the TetA pump played an important role in tigecycline efflux with AdeABC and AdeIJK acting in a synergistic and/or additive manner.

Mutations in the *tet*(A) gene have been associated with reduced tigecycline susceptibility in carbapenem-resistant *K. pneumoniae* (CRKP) (Chiu et al., 2017; Xu et al., 2021b; Peng et al., 2022b). Sixteen CRKP strains were found to have individual (81%) or combination (63%) mutations in *ramR* and *tet*(A) (Chiu et al., 2017). Through the complementation into the *tet*(A)/*ramR*-deficient strains, they discovered a notable elevation in tigecycline MICs in *tet*(A) frame-shift mutants complementary strains, meanwhile, a synergistic effect of resistance was observed in strain without *ramR* but a mutated *tet*(A) compared to the wild-type strain overexpressing the mutated *tet*(A). This implied that mutations in *ramR* and *tet*(A) might be the primary mechanism of tigecycline resistance and act synergistically. Another study performed whole genome sequencing of 63 CRKP isolates and chose isolates with *tet*(A) to conduct tigecycline resistance screening under selective pressure (Xu et al., 2021b). They identified that 71.4% of the tigecycline non-susceptible strains were found to have *tet*(A) mutations and exhibit high-level tigecycline resistance. These amino acid substitutions were confirmed in *E. coli* as the overexpression strains showed a 2-8-folds increase in tigecycline MICs.

The tigecycline resistance caused by *tet*(A) and *ramR* mutations is also present in *S. enterica*. Hentschke et al. discovered a *tet*(A) mutant that was located on a plasmid with Tn*1721* in tigecycline resistance *S. enterica* and increased tigecycline MIC when transferred to *E. coli* (Hentschke et al., 2010a). The isolate also possessed a frame-shift mutation in *ramR* and the overexpression of the mutants did not affect tigecycline susceptibility compared to the increase when overexpressed wild-type *ramR.* It indicated that the resistance might related to the enhanced efflux of AcrAB through the up-regulation of RamA resulting from *ramR* mutation. Together with *tet*(A) mutation, these two mechanisms mediating low-level resistance may act synergistically, leading to high-level tigecycline resistance. The same synergetic effect has been identified in tigecycline non-susceptible *S. enterica* with *tet*(A) frame-shift mutation (Akiyama et al., 2013). This confirmed that *tet*(A) could decrease sensitivity to tigecycline at a low level, with *ramR* inactivation acting as an additional resistance mechanism that might confer high-level resistance.

Another MFS efflux pump, Tet(Y), has also been reported to be associated with tigecycline resistance in *A. baumannii* (Wang et al., 2021c). Whole-genome sequencing identified an isolate without any known tigecycline resistance genes and mutations but carried a novel MFS efflux pump-encoding gene, *tet*(Y) on its plasmid. The overexpression of *tet*(Y) and *tet*(Y) plasmid could increase the tigecycline MICs, indicating that *tet*(Y) is related to tigecycline susceptibility reduction. The adjacent Tn*5393* in its genetic background suggested that resistance caused by the novel plasmid carrying *tet*(Y) might be transmitted between isolates.

Tet(L), an MFS efflux pump often reported in Gram-positive bacteria, has been discovered in Gram-negative bacteria recently. Sun et al. identified *tet*(L) in tigecycline-susceptible *Moraxella catarrhalis*, implying that it does not affect tigecycline resistance in *M. catarrhalis* (Sun et al., 2021). A variant of *tet*(L) was identified in the chromosomes of *Campylobacter* spp. with a prevalence rate of approximately 17%, of which the overexpression revealed a 4-fold increase in MIC (Yao et al., 2020). Genetic environment analysis revealed that the *tet*(L) variant was located in a genomic island with IS*1216E* and other resistance genes inserted into the conserved gene *potB*, suggesting that the variant might not only mediate resistance but also spread through horizontal gene transfer.

### 2.7 Other efflux pump

Except for the aforementioned common efflux pump families, novel efflux pumps associated with tigecycline resistance continue to be identified. An efflux pump, KpgABC, was identified in *K. pneumoniae*, which was associated with tigecycline nonsusceptibility (Nielsen et al., 2014). Following an increase in tigecycline MIC from 1 mg/L to 4 mg/L in clinical strains collected before and during patient tigecycline treatment, and no increase in the expression of known efflux pump-associated genes as revealed by RT-qPCR studies, whole-genome sequencing was performed to identify putative novel resistance genes. An IS*5* insertion in the upstream of a putative efflux pump, which was named KpgABC, was identified. A 4-fold increase in MIC after overexpression of *kpgABC* verified its role in the reduction of tigecycline sensitivity.

## 3 Resistance mechanisms associated with antibiotic modification

*tet*(X) genes encode a flavin-dependent monooxygenase that modifies tetracyclines, rendering them inactive. It is capable of inactivating all tetracycline antimicrobial agents, including tigecycline and eravacycline, while mobile genetic elements like transposons often mediate its spread (Anyanwu et al., 2022). The *tet*(X) genes that have been identified to date include *tet*(X), *tet*(X1-X15), and *tet*(X18-X47), with the distribution illustrated in **Table 3**. *tet*(X), *tet*(X1) and *tet*(X2) are only vertically transmitted in the environment and microbiota, conferring low levels of resistance to tigecycline, with *tet*(X1) and *tet*(X2) shared 66% and 99% identity with *tet*(X) (Whittle et al., 2001; Yang et al., 2004; Hassan et al., 2018). In recent years, plasmid- or chromosome-localized *tet*(X3-X6) has been identified as a significant factor contributing to high tigecycline resistance and observed in a diverse range of strains.

**Table 3:** Specific information on *tet*(X) and its variants.

	Species	Localization	PMID
*tet*(X)	*Bacteroides fragilis*	plasmid/chromosome	15452119/22014885
*tet*(X1)	*Bacteroides/Enterococcus* spp.	chromosome	11472924/29274469/22014885
*tet*(X2)	*Bacteroides/Empedobacter stercoris/Acinetobacter pittii*	chromosome	11472924/32731802/37141282
*tet*(X3)	*Acinetobacter* species	chromosome/plasmid	33287863/36409072/32816739/35124286/34851156/34987485/36081799
*tet*(X4)	*Escherichia coli/Acinetobacter species/Klebsiella pneumoniae/Klebsiella quasipneumoniae/Citrobacter braakii/Citrobacter freundii/Shewanella xiamenensis/Escherichia fergusonii/Salmonella enterica*	plasmid/chromosome	33287863/32345737/33992939/31235960/36016796/32846111/36326874/32853333/34431695/31429665/34937176/35138117/35021125/35793774/34899627/32816739/34987485/
*tet*(X5)	*Acinetobacter* species	plasmid	31611352/33373881/34801490/33347964
*tet*(X6)	*Acinetobacter* species/*Myroides phaeus/Proteus* spp.*/Chryseobacterium indologenes/Providencia rettgeri/Ignatzschineria indica/Oblitimonas alkaliphila/Escherichia coli*	chromosome/plasmid	34680819/33373881/35124286/34987485/35966843/32345737/34801490/33762210/34851156/36081799/32766775/33559156/34936926/32068864
*tet*(X7)	*Escherichia coli*	plasmid	33820767
*tet*(X7-13)	human commensal metagenomes	NA	32415166
*tet*(X14)	*Empedobacter stercoris/Riemerella anatipestifer*	chromosome	32731802
*tet*(X15)	*Acinetobacter variabilis*	chromosome	34109404/34987485
*tet*(X18-44)	*Riemerella anatipestifer*	chromosome/plasmid	34098588
*tet*(X45-47)	*Bacteroidaceae/Enterococcaceae/Candidatus Melainabacteria*	NA	34935428
unnumbered *tet*(X) variant	*Klebsiella aerogenes/Empedobacter falsenii/Riemerella anatipestifer*	plasmid	34346701/31778164/35944374

NA, not applicable.

Plasmid-borne *tet*(X3) and *tet*(X4) genes were initially identified in tigecycline-resistant *A. baumannii* and *E. coli* from Chinese edible animals and the environment in 2019, exhibiting 85.1% and 94.3% identity to *tet*(X), respectively (He et al., 2019). The antimicrobial susceptible assay revealed that the overexpression of *tet*(X3) and *tet*(X4) in *E. coli* significantly elevated the tigecycline MICs. Furthermore, the *in vivo* results demonstrated that *tet*(X3) and *tet*(X4) might also affect the tigecycline treatment in the mouse infection model, leading to clinical treatment failure. The genetic background analysis presented that they were both adjacent to the IS*Vsa3* on their respective plasmids, suggesting the possible transmission between strains. The article also conducted a retrospective analysis and discovered that *tet*(X3) and *tet*(X4) are already prevalent in clinical isolates, indicating that the *tet*(X) variants are emerging as one of the most important tigecycline resistance genes.

*tet*(X3) and *tet*(X4) genes were identified in a range of isolates. A screening of *tet*(X) variants of *Acinetobacter* species from pig, bird, and human sources in China identified 193 *tet*(X3)- or *tet*(X4)-positive *Acinetobacter* species (5.0%), of which 188 carried *tet*(X3) and 5 carried *tet*(X4) (Chen et al., 2020). Seven novel *tet*(X3) variants were discovered in tigecycline-resistant *Acinetobacter* species, sharing 15.4%-99.7% amino acid identity with Tet(X3). Only *tet*(X3.7) and *tet*(X3.9) could increase the tigecycline MIC and were found to be in proximity to mobile genetic elements and site-specific recombinase *xerD* (Cheng et al., 2022). *tet*(X3) identified on *Acinetobacter* plasmids are often found in proximity to mobile genetic elements, including IS*Vsa3* (IS*CR2*), IS*4*, and IS*26*, which facilitate its transfer between strains (Zhang et al., 2020; Cheng et al., 2021b; Cheng et al., 2022; Wang et al., 2022b). Other reports have documented that the majority of *tet*(X4) localized on *E. coli* plasmids are commonly surrounded by IS*Vsa3* (IS*CR2*), while also finding the presence of *tet*(X4) and *mcr-1* co-occurrence plasmid as well as the chromosomally located *tet*(X4) in *E. coli* (Chen et al., 2019; Sun et al., 2019; Ding et al., 2020; Li et al., 2020; Lv et al., 2020a; Li et al., 2021b; Mohsin et al., 2021; Chen et al., 2023; Wang et al., 2022a). The identifications of *tet*(X4) in *E. coli*, *K. pneumoniae*, *K. quasipneumoniae*, *Citrobacter braakii*, and *C. freundii* have been reported in the presence of four core genetic backgrounds, all of which are adjacent to IS*Vsa3* (IS*CR2*) or IS*26* (Li et al., 2021a; Zhai et al., 2022). Dao et al. has discovered *tet*(X4) in tigecycline- and carbapenem-resistant *Shewanella xiamenensis* (Dao et al., 2022). It was co-located with *bla*_OXA-48_ on the plasmid and flanked by IS*91* family transposase genes, indicating that its acquisition might be mediated by mobile genetic elements. In *E. fergusonii*, *tet*(X4) was found in co-occurrence with *bla*_TEM-1B_ and *floR* on a mobile plasmid that was highly homologous to plasmids from *E. coli, E. cloacae, and Klebsiella* spp. (Guan et al., 2022). Moreover, *tet*(X4) was identified in an extensively drug-resistant *Salmonella enterica* (Abd El-Aziz et al., 2021).

A novel plasmid-mediated tet(X) variant, *tet*(X5), was reported in 2020 in a tigecycline resistant *A. baumannii* (Wang et al., 2019). Tet(X5) exhibits amino acid identity with Tet(X3) and Tet(X4) at 84.5% and 90.5% with a similar binding site and comparable affinities for tetracyclines, respectively. The overexpression of *tet*(X5) demonstrated an increase in the MICs of tetracyclines, yet the level of Tet(X5)-mediated tigecycline resistance was slightly lower in comparison to the high-level resistance mediated by Tet(X3/4), as previously reported. A comparable genetic context to that of *tet*(X3/4) was also identified in *tet*(X5), suggesting that *tet*(X) variants might disseminate through IS*Vsa3*. Other reports on *tet*(X5) in *A. baumannii* have implied that it is situated within the IS*Vsa3* (IS*CR2*)-mediated *tet*(X) transposon structure, thereby increasing its transmission risk between the environment and the clinic (Chen et al., 2021). *tet*(X5) has been also identified in other resistant *Acinetobacter* species (Dong et al., 2022a), Tang has found it co-located with *bla*_NDM-3_ in *A. indicus* plasmid that mediates tigecycline resistance in the strain (Tang et al., 2021).

*tet*(X6) was initially identified on the chromosome of *Myroides phaeus* with the overexpression strains revealing only a 2-4-fold increase in tetracyclines MICs, while its similar adjacency to IS*Vsa3* might contribute to the transmission (Liu et al., 2020). The reason for its mediation of lower levels of resistance may be attributed to the lower tetracycline-binding capacity of Tet(X6) in comparison to other Tet(X) variants. Further retrospective analysis revealed that *tet*(X6) was also found in various Proteus spp. and *Acinetobacter* species. Many reports have identified *tet*(X6) variants on the chromosome of *A. baumannii*, *Acinetobacter* species, *Chryseobacterium indologenes*, *Providencia rettgeri*, *Ignatzschineria indica*, and *Oblitimonas alkaliphile* (Li et al., 2020; Chen et al., 2021; Hsieh et al., 2021; Li et al., 2021d; Damas et al., 2022; Dong et al., 2022a; Wang et al., 2022b). Additionally, plasmid-localized *tet*(X6) genes have been detected on tigecycline-susceptible *A. towneri* plasmid with a genetic background also associated with IS*Vsa3* (IS*CR2*) (Cheng et al., 2021a). The detection of *tet*(X6) in a susceptible plasmid indicates the potential for cryptic spread of this novel plasmid-mediated tigecycline resistance. The majority of reported *tet*(X6) genes carried by plasmids from *Acinetobacter* species are located adjacent to IS*Vsa3* (IS*CR2*) or IS*Aba1* and often co-localized with *tet*(X3), *bla*_OXA-58_ or other resistance genes, with the plasmid conjugates presented increased tigecycline MICs (Zheng et al., 2020; Cheng et al., 2021b; Li et al., 2021d; Chen et al., 2022). Xu and Usui et al. also identified *tet*(X6) in plasmids from tigecycline-resistant *E. coli*, which was co-located with *mcr-1* in the hotspot of resistance genes, in proximity to a variety of mobile genetic elements such as Tn*As1*, Tn*As3*, and IS*Vsa3* (Usui et al., 2022; Xu et al., 2021a).

In addition to the above *tet*(X) variants commonly reported about tigecycline resistance, other variants have also been discovered to mediate resistance. A plasmid from a tigecycline-resistant *E. coli* strain was found to contain *tet*(X7) with the co-occurrence of *mcr-1.1* (Soliman et al., 2021). The tigecycline MIC was significantly elevated after plasmid conjugation, while *tet*(X7) was adjacent to IS*CR3* which might play a role in the transmission of resistance. *tet*(X14) was identified on tigecycline- and colistin-resistant *Empedobacter stercoris*, which exhibited 67.14%-96.39% sequence identity with other variants (Cheng et al., 2021a). It was co-localized with *tet*(X2) on the chromosome and the overexpression of *tet*(X14) resulted in a significant reduction in the tigecycline susceptibility. With no mobile genetic elements detected in its vicinity, *tet*(X14) might be a heterologous gene obtained by recombination. Further screening in Genbank revealed that only *Riemerella anatipestifer* carried *tet*(X14), leading to the presumption that the *Flavobacteriaceae* are its reservoir. *tet*(X15) was discovered on the chromosome of a tigecycline-resistant *A. variabilis*, with overexpression strains exhibiting elevated tigecycline MICs, suggesting that *tet*(X15) contributes to reduced tigecycline susceptibility (Li et al., 2021c; Li et al., 2021d). Genetic background analysis indicated that it was located within the IS*Aba1*-binding complex transposon Tn*6866* and that IS*Aba1* might promote the spread of *tet*(X15).

*R. anatipestifer* has been reported as a probable source of the *tet*(X) gene. A tracking screen for *tet*(X) in public databases revealed that it appeared as early as the 1960s in *R. anatipestifer* and was the primary *tet*(X) vector during the initial stages. Comparative genomic analysis indicated that *tet*(X) variants were likely produced through the dissemination of *tet*(X) between *Flavobacteriaceae* and *E. coli*/*Acinetobacter* species, with IS*CR2* playing a pivotal role, leading to the hypothesis that *R. anatipestifer* might be a potential natural source of *tet*(X) (Zhang et al., 2021b). Various *tet*(X) variants, including *tet*(X18-X44), were identified on the chromosome and plasmid of *R. anatipestifer*, and the overexpression strains revealed that most of these variants conferred tigecycline resistance to *E. coli*, while the less frequently occurring *tet*(X27/29/30) variants might be recessive or silent (Umar et al., 2021; Zhu et al., 2022). Further analysis implied that these variants were seldom found adjacent to mobile genetic elements, which lent support to the hypothesis that *R. anatipestifer* is a natural source of *tet*(X). Zhang et al. have screened the human microbiome samples and identified three novel *tet*(X) variants, designated *tet*(X45), *tet*(X46), and *tet*(X47), which were found to mediate high levels of tigecycline resistance (Zhang et al., 2021b). The macrogenomic analysis suggested that *tet*(X) variants were predominantly derived from *Bacteroidaceae* of the human gut, with IS*Bf11* and IS*4351* being the most likely to mediate the spread. The naming rules for *tet*(X) variants are somewhat disorganized, and there is a tendency for duplicate nomenclature to occur due to the time at which studies are reported. Consequently, many studies have uniformly referred to newly discovered *tet*(X) variants as *tet*(X) variants, rather than numbering them. The above are organized according to the nomenclature that was in use at the time of reporting in the literature. Novel *tet*(X) variants have been reported on plasmids of *K. aerogenes* and *E. falsenii* and both of these variants mediated high-level tigecycline resistance, while the *tet*(X) variant in *K. aerogenes* was co-localized with another tigecycline resistance determinant cluster, *tmexCD3-toprJ3*, on a novel plasmid (Zeng et al., 2020; Hirabayashi et al., 2021).

## 4 Resistance mechanisms associated with antibiotic binding

### 4.1 Ribosome-related gene mutations

An amino acid substitution mutation, V57L, in the ribosomal small subunit constitutive protein S10 encoding gene *rpsJ* has been identified in tigecycline resistant *K. pneumoniae*, which has been previously reported to relate to tetracycline resistance in *Neisseria gonorrhoeae* (Villa et al., 2014). This mutation is located at the tip of a conserved flexible loop consisting of amino acids 53-60 in the S10 ribosomal protein and this region is near the tigecycline target site and is associated with the ribosome binding to tigecycline. The article postulated that the mutation in *rpsJ* affected tigecycline susceptibility by altering the ribosome structure near the tigecycline-binding site or interfering with the coordination of Mg^2+^ ions, which resulted in reduced tigecycline binding to the 16S rRNA therefore reduced the tigecycline susceptibility. Mutations in *rpsJ* at V57 locus were identified in all resistant isolates through tigecycline resistance screening of *E. coli*, *K. pneumoniae*, and *A. baumannii*, as well as in another report about tigecycline resistant *K. pneumoniae* from clinical source and laboratory evolution (Beabout et al., 2015; Fang et al., 2016). Other *rpsJ* mutations, predominantly V57L amino acid substitutions, have been documented in *S. maltophilia*, *K. pneumoniae*, *E. coli*, and *A. baumannii* with the mutations reducing tigecycline susceptibility by affecting the structure of ribosomal protein S10 (Villa et al., 2014; Hammerstrom et al., 2015; Li et al., 2016a; He et al., 2018; Blanco et al., 2019; Xu et al., 2020; Zhang et al., 2021a). The overexpression of corresponding mutations in *E. coli* revealed that the majority of amino acid substitutions (V57L, V57D, and V57I) resulted in a modest elevation in tigecycline MICs, while the most pronounced increase was observed in V57L overexpression (Izghirean et al., 2021). However, none of these mutations were as significant as other resistance determinants, such as efflux pumps, on tigecycline susceptibility, suggesting that *rpsJ* mutation in conjunction with other resistance mutations or determinants is necessary to mediate high-level tigecycline resistance.

A report about a tigecycline resistant *A. baumannii* has found an amino acid substitution mutation in *rrf*, the gene encoding the ribosomal recycling factor RRF, with a slight reduction in tigecycline MIC after the complementation of wild-type *rrf* into the isolate possessed mutated *rrf* (Hua et al., 2021). Subsequent transcriptome analysis demonstrated that the expression levels of various genes associated with ribosome regulation, energy production, biosynthesis, and transportation increased. The western blotting and polysome profiling revealed that *rrf* mutants displayed a reduction in RRF expression and an accumulation of 70S ribosomes, suggesting that the mutation in *rrf* affected the presumed function of RRF in dissociating and recycling tigecycline-bound ribosomes, and at the same time decreased tigecycline’s binding affinity to the ribosomal A-site, leading to a decrease in tigecycline susceptibility. Hammerstrom et al. have discovered other *rrf* amino acid substitutions, deletion mutations, and upstream mutations in tigecycline resistant *A. baumannii* obtained through evolution under antibiotic stress (Hammerstrom et al., 2015). *rrf* mutation has also been identified in a tigecycline resistant *S. maltophilia* obtained under tigecycline pressure, suggesting that it might play a role in adaptation to tigecycline (Blanco et al., 2019).

In addition to the two ribosome-associated protein mutations previously discussed, mutations in the 30S ribosomal protein S21 encoding gene *rpsU* and in the ribosomal protein S1 encoding gene *rpsA* have been reported as potential contributors to tigecycline resistance in *S. maltophilia* (Hammerstrom et al., 2015).

### 4.2 Ribosomal protection proteins-related mutations

It has been reported that tigecycline can maintain the binding to 16S rRNA in the presence of the ribosomal protection protein Tet(M) due to the C9-glycyl substituent hinders access of Tet(M) binding to the ribosome, which fails the ribosomal protection (Arenz et al., 2015). This ribosomal protection protein is frequently identified in tigecycline resistant Gram-positive bacteria, whereas among Gram-negative bacteria, it has only been reported to be associated with low tigecycline susceptibility in *N. gonorrhoeae* (Zhou et al., 2022). The *tet*(M) gene was carried by the plasmid of *N. gonorrhoeae* and the correlation analysis revealed that the carriage was significantly correlated with low tigecycline susceptibility (Zhou et al., 2022). Linkevicius et al. have found that *tet*(M) mutations may result in reduced tigecycline susceptibility (Linkevicius et al., 2015). They overexpressed *tet*(M) in *E. coli* and constructed mutant libraries for resistance screening that discovered 13 mutations that could cause increased MICs, in which L505 deletion mutation and the Q620R/S310P mutation combined with S508A significantly elevated tigecycline MICs. Furthermore, the Q620R/S310P combined with S508A caused the most significant MIC elevation. These mutations may contribute to tigecycline susceptibility reduction by affecting the structure and function of Tet(M).

A study about tigecycline resistance *A. baumannii* screening under antibiotic stress has found a frame-shift in S-adenosylmethionine (SAM)-dependent methyltransferase encoding gene *trm* due to the deletion mutation, which caused the truncation of the protein and reduced the susceptibility (Chen et al., 2014). The complementation of wild-type *trm* resulted in the restoration of susceptibility to minocycline, doxycycline, and tigecycline, indicating that the *trm* mutation might cause the isolate to become resistant to tetracyclines. Methyltransferases serve to safeguard the host genome from foreign DNA and play a vital role in epigenetic regulation and antibiotic resistance. They postulated that *trm* mutations may facilitate the emergence of tigecycline resistance by interfering with the ribosomal protein methylation. Many other tigecycline resistance-associated amino acid substitutions and deletion mutations in the *trm* gene have been documented in *A. baumannii* (Trebosc et al., 2016; Ghalavand et al., 2022).

The *rpoB* gene, which encodes the β subunit of DNA-dependent RNA polymerase, is evolutionarily conserved. Hua et al. have found a G136D amino acid substitution in *rpoB* from a tigecycline resistant *A. baumannii* (Hua et al., 2021). The Raman spectroscopy demonstrated that the overexpression of the mutation *rpoB* markedly enhanced the isolate’s tolerance to tigecycline, although it only induced a minimal reduction in tigecycline susceptibility. Further transcriptome analysis revealed that multiple transcriptional regulatory genes potentially implicated in stress response and drug resistance exhibited either increased or decreased expression, with a decreased expression of *trm* and a gene encoding an AcrR/TetR regulatory protein, indicating that the mutant *rpoB* might be involved in the regulation of *trm* expression and the transcriptional regulatory genes, thereby conferring resistance to tigecycline.

## 5 Resistance mechanisms associated with membrane

### 5.1 Membrane permeability-related mutations

During the tigecycline resistance screening under antibiotic stress, a resistant *A. baumannii* isolate was generated, wherein a frame-shift mutation was identified in glycerol-3-phosphate acyltransferase encoding gene *plsC*, resulting in truncation of the protein (Li et al., 2015). The complementation of the wild-type *plsC* gene recovered the tigecycline MIC reduction, suggesting that *plsC* was related to decreased tigecycline susceptibility. The alterations in membrane potential were quantified and it implied that the mutant exhibited the highest membrane potential, while it decreased after the wild-type gene complementation, indicating that the mutation might influence the membrane permeability. They speculated that the *plsC* mutation mediated the tigecycline resistance primarily by affecting the phospholipid synthesis, altering the membrane, and, consequently, increasing the tigecycline permeability.

A mutation in the C13 family peptidase encoding gene *abrp* has been identified in tigecycline resistant *A. baumannii*, and the truncation of the protein might be associated with tigecycline resistance (Li et al., 2016b). They demonstrated that the *abrp* knockout resulted in reduced susceptibility to tigecycline and increased cell membrane permeability in the isolate, whereas complementation with wild-type *abrp* restored both susceptibility and cell membrane permeability. These findings implied that *abrp* deletion might affect the membrane permeability and consequently impact the tigecycline susceptibility.

He et al. conducted tigecycline resistance screening in the *acrAB* knockout and wild-type *E. coli* isolates and obtained resistant mutants (He et al., 2016). Sequencing analysis revealed that a 2-amino acid deletion in phospholipid translocation-related gene *mlaA* of the ATP-binding cassette transporter (ABC) transport system was presented in both the knockout and wild-type isolates. This mutation truncated and inactivated the MlaA protein. The tigecycline MICs of *mlaA*-absent strains remained unchanged, while the complementation strains demonstrated an 8-fold increase in tigecycline MIC. It led to the hypothesis that the mutation in *mlaA* might enhance phospholipid transfer from the outer to the inner membrane, thereby strengthening the outer membrane barrier and contributing to the resistance. Furthermore, the article identified mutations in *marR* and *rpsJ* after the *mlaA* mutation, suggesting that multiple resistance mechanisms can accumulate during the development of tigecycline resistance.

### 5.2 Membrane structure-related mutations

In another study of tigecycline resistance induced through antibiotic pressure in *A. baumannii*, mutations in UDP-N-acetylglucosamine dehydrogenase encoding gene *gna* and ABC-transporter encoding gene *msbA* have been identified in the obtained resistant strains and postulated to be associated with tigecycline resistance (Hammerstrom et al., 2015). *gna* is situated within the K motif, which encodes extracellular polysaccharide biosynthetic enzymes. This enzyme plays a role in the assembly of capsule or lipooligosaccharide (LOS). It is hypothesized that a frame-shift mutation in *gna* may inactivate the protein and cause structural changes in the capsular polysaccharide or LOS, affecting the rate of diffusion of tigecycline into the cell. MsbA functions as a transporter protein that facilitates the transfer of lipid A from the medial leaflet to the periplasmic side of the inner membrane. As the majority of the observed mutations are concentrated in their substrate-recognition and transmembrane regions, they postulate that mutations may enhance the specificity of the pump, thereby inducing the tigecycline efflux.

An IS*Aba16* insertion mutation in *gnaA* was also identified in high-level resistant *A. baumannii* (Xu et al., 2019). Even though they did not validate the function of the gene, the absence of any other tigecycline resistance-associated genes or mutations in the isolate may also corroborate the hypothesis that the *gnaA* mutation was correlated with resistance. In a separate study, mutations in *tviB*, the resistance-related gene encoding the UDP-N-acetylglucosamine dehydrogenase, were identified in *A. baumannii* (Lucaßen et al., 2021a). This study revealed a high degree of TviB amino acid sequence diversity in both resistant and susceptible isolates, in which a seven amino acid insertion variant might be relevant to tigecycline resistance. The presence of sequence diversity suggests that we need to be cautious in interpreting mutations in *tviB* and that further characterization is required.

The screening of tigecycline resistance in *S. maltophilia* also discovered mutations in genes encoding enzymes related to lipopolysaccharide (LPS) biosynthesis and phosphatidic acid biosynthesis, which may relate to tigecycline resistance (Blanco et al., 2019). The mutated genes included the phosphoethanolamine transferase encoding gene, lipid A biosynthesis lauroyl acyltransferase encoding gene *htrB*, the UDP-glucose dehydrogenase encoding gene *ugd*, and the diacylglycerol kinase encoding gene *dgkA*. They hypothesized that the mutations led to impacts on phospholipid and LPS synthesis, modifications of the bacterial outer membrane, preventing the uptake of tigecycline, and therefore increased the resistance to tigecycline.

Inactivation of the TolC-like outer membrane protein AbuO has also been reported in correlation to tigecycline resistance in *A. baumannii* (Srinivasan et al., 2015). Srinivasan et al. demonstrated a notable reduction in tigecycline MIC in *abuO* knockout strains, and the RT-qPCR analysis indicated that the expression of efflux pump genes like *acrD*, and regulatory genes like *baeR* was elevated. MerR-type transcriptional regulator SoxR binding to *abuO* promoter revealed that *abuO* in *A. baumannii* was regulated by SoxR.

## 6 Resistance mechanisms associated with DNA repair

In addition to the aforementioned resistance mechanisms, the RecA and RecBCD pathways, which are involved in the regulation of DNA damage induction in *A. baumannii*, have also been reported to be associated with tigecycline resistance (Ajiboye et al., 2018). The knockout of *recA*, a homologous recombinase encoding gene involved in DNA damage repair, resulted in increased tigecycline susceptibility. Similarly, the knockout of *recB*, *recC*, and *recD*, genes playing a crucial role in repairing antimicrobial-induced bacterial oxidative DNA damage, led to a relative increase in susceptibility. The involvement of the RecA-RecBCD pathway in tigecycline resistance might be related to the reduced ability of deletion mutant strains to repair DNA.

## 7 Concluding remarks

**Figure 1:**
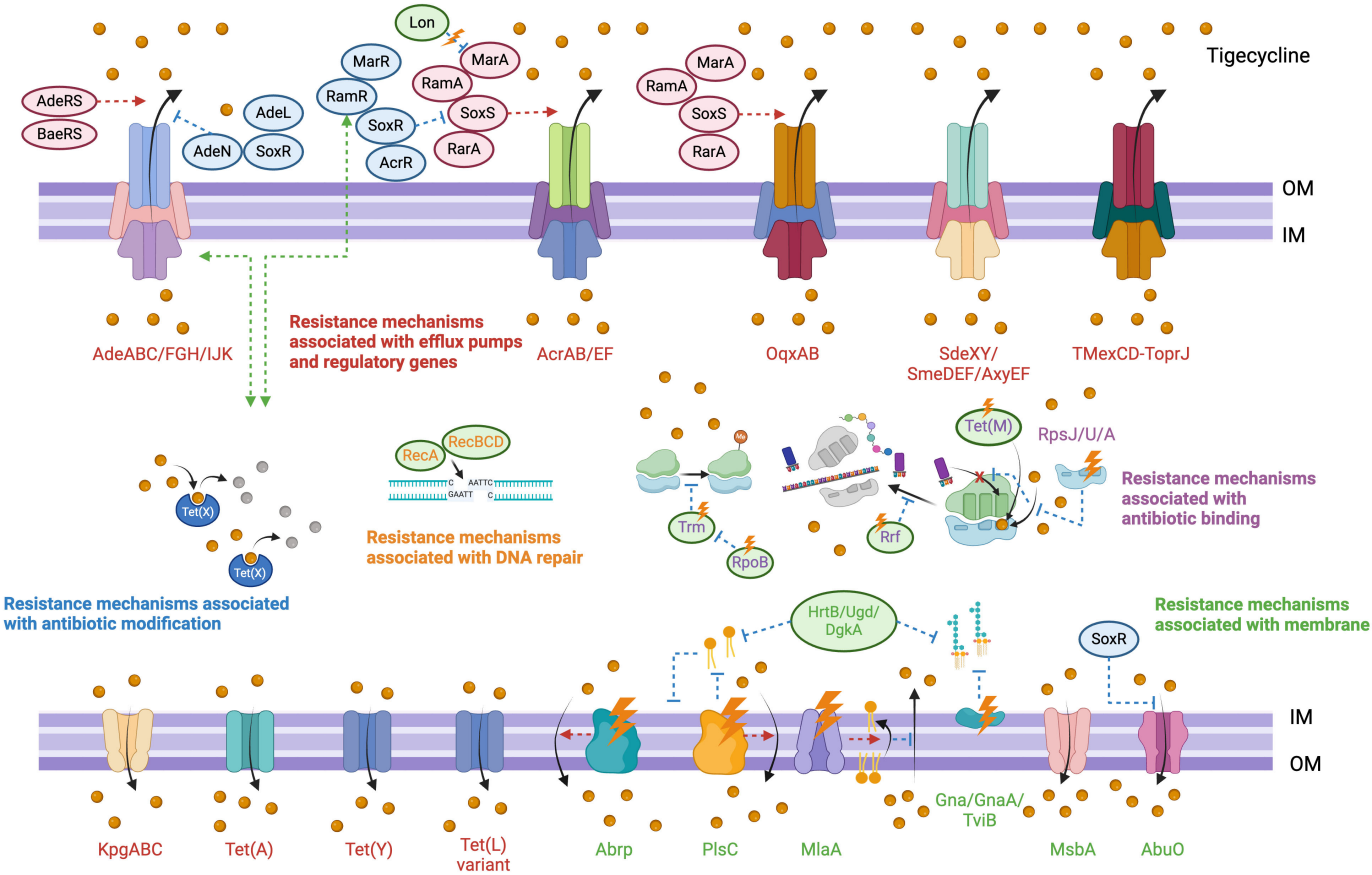
Mechanisms and the regulators of tigecycline resistance in Gram-negative bacilli. OM, outer membrane; IM, inner membrane; red arrow, positive regulation; blue arrow, negative regulation; green arrow, synergistic effect.

Tigecycline, revered as the ‘last line of defense’ against multidrug-resistant bacterial infections, serves as a cornerstone antibiotic in clinical practice. Unraveling the mechanisms underpinning tigecycline resistance is paramount for the antimicrobial resistance prevention. Current insights into resistance mechanisms in Gram-negative bacilli predominantly revolve around efflux pumps and antibiotic modification mechanisms that either expel the drug from the isolates or render it inactive. While less reported, resistance mechanisms impacting drug binding and membrane permeability often exert simultaneous effects on bacterial biosynthesis, as illustrated in **Figure 1**.

Notably, numerous tigecycline resistance genes or determinants, such as TMexCD-TOprJ pumps and *tet*(X) variants, cluster near mobile genetic elements, signaling an escalating risk of tigecycline resistance dissemination. Attention must be directed towards dissecting the interplay between resistance genes and mobile genetic elements to curb the cross-transmission of tigecycline resistance genes across clinical and environmental spheres. Moreover, certain resistance genes or determinants have been implicated in altering various phenotypes other than tigecycline resistance. The disruption of *gnaA*, gene related to the capsular polysaccharide synthesis in *A. baumannii*, can affect the tigecycline resistance as well as the pathogen morphology and virulence through changing the membrane composition (Xu et al., 2019). AcrAB efflux pump can efflux not only tigecycline but also antibacterial molecules such as bile, mammalian steroid hormones, and antimicrobial peptides, which allows them to survive better in the host (Lister et al., 2012). After tigecycline treatment in a patient, mucoid strains resistant to tigecycline were isolated, accompanied by decreased serum tolerance, enhanced biofilm formation ability, and reduced virulence in *Galleria mellonella* (Zhang et al., 2022). Tigecycline resistant genes or mutations may have collateral effects beyond resistance, such as altered virulence or morphology of the bacteria.

This study offers a comprehensive overview of potential resistance mechanisms to tigecycline in Gram-negative bacilli, elucidating the intricacies and diversities of resistance mechanisms across different species. Furthermore, it also lays the groundwork for preempting tigecycline resistance and identifying fresh avenues for tigecycline-resistant therapies. Delving into new tigecycline resistance mechanisms and conducting detailed explorations of known pathways are imperative to proactively address potential public health crises stemming from antibiotic resistance.
